# Partial Validation of the Dutch Model for Emission and Transport of Nutrients (STONE)

**DOI:** 10.1100/tsw.2001.262

**Published:** 2001-11-17

**Authors:** G.B.J. Overbeek, A. Tiktak, A.H.W. Beusen, P.J.T.M. van Puijenbroek

**Affiliations:** National Institute of Public Health and the Environment (RIVM), Bilthoven, The Netherlands

**Keywords:** environmental modeling, validation, nutrient emissions, nitrate, phosphate, scale issues

## Abstract

The Netherlands has to cope with large losses of N and P to groundwater and surface water. Agriculture is the dominant source of these nutrients, particularly with reference to nutrient excretion due to intensive animal husbandry in combination with fertilizer use. The Dutch government has recently launched a stricter eutrophication abatement policy to comply with the EC nitrate directive. The Dutch consensus model for N and P emission to groundwater and surface water (STONE) has been developed to evaluate the environmental benefits of abatement plans. Due to the possibly severe socioeconomic consequences of eutrophication abatement plans, it is of utmost importance that the model is thoroughly validated. Because STONE is applied on a nationwide scale, the model validation has also been carried out on this scale. For this purpose the model outputs were compared with lumped results from monitoring networks in the upper groundwater and in surface waters. About 13,000 recent point source observations of nitrate in the upper groundwater were available, along with several hundreds of observations showing N and P in local surface water systems. Comparison of observations from the different spatial scales available showed the issue of scale to be important. Scale issues will be addressed in the next stages of the validation study.

## INTRODUCTION

In the Netherlands, agriculture is the dominant source of nutrients in groundwater and surface water. Nutrient excretion by intensive animal husbandry in combination with fertilizer use is the main cause of these nutrient emissions. Currently, stricter eutrophication abatement policies, which include high levies and mandatory manure trade contracts, are in development by the Dutch government. These policies should lead to the realization of national and European Community (EC) targets for reduction of nutrient losses to the environment and compliance with the EC nitrate directive. The STONE model has been developed by several Dutch research institutes to evaluate current and future policy measurements. Consisting of three submodels, which have been validated independently, the STONE model calculates the emission of N and P to groundwater and surface waters in the Netherlands on a nationwide scale.

Because of the potentially severe economic, environmental, social, and jurisdictional impact of eutrophication abatement plans, it is important to use a validated model. Following improvement of the biophysical characterization of the Netherlands[1] and the continued testing of the submodels, a comprehensive validation study of the whole system was launched early in 2001. The validation study consists of several stages. In the first stage, the model is compared with lumped observations from both groundwater- and surface-water-monitoring networks. This stage offers general information about model behavior vs. real-world data at an aggregated national level and will also indicate whether the model can reproduce frequency distributions of observed concentrations. During the following stages of the model validation, the focus will be on underlying processes, spatial patterns, temporal dynamics, and model uncertainty. This paper only reports results from the first stage of the study.

## DATA AND METHODS

### Model Description

The STONE model represents the combined effort of several research institutes in the Netherlands. The aim of STONE is to predict the current and future emissions of N and P to surface water and groundwater. The model (version 2.0) consists of three submodels (Fig. 1). First, the CLEAN submodel calculates the distribution of manure across the Netherlands and the emission of nutrients to soil and air[2]. Second, the OPS/SRM submodel calculates the deposition of N resulting from agricultural and secondary sources[3]. Finally, the ANIMO submodel[4], which is incorporated in a geographical shell GONAT[5], computes the transport of nutrients to both groundwater and surface water. In ANIMO as it is used in STONE, the crop uptake is calculated by the (QUADMOD) module[6]. [Au: need to define acronyms?]

The chain of models is accessed by a graphical user interface, which allows the user to manipulate the model scenarios, run the model, and view the model outputs. STONE is provided with a large set of fixed data, e.g., hydrological data, which are calculated with the SWAP model[7]. These fixed data are considered boundary conditions for STONE. Because spatial and temporal resolutions of the three submodels are different, conversion programs have been included.

The CLEAN model requires input and generates results for 31 agricultural regions in the Netherlands. In each of these regions, six crop types and seven soil types are distinguished. The manure production is calculated from 42 categories of animals and manure excretion numbers for each animal. The regional distribution of manure is controlled both by costs of transport and costs of penalties for exceeding manure standards.

The OPS/SRM model computes the deposition of N on the basis of a 5 × 5 km grid.

The spatial resolution for GONAT/ANIMO consists of 6405 homogeneous areas (STONE plots), which vary in size from 25 to about 20,000 ha. These areas are based on a classification (250- × 250-m grid) of land use, soil type, and hydrological and soil chemical characteristics.

Simulations start on 1 January 1986. Because the hydrological data are available for 15-year periods, all scenarios follow a simulation period of multiples of 15 years. Two hydrological data sets are available, one for the period 1971 to 1985 and one for the period 1986 to 2000. The hydrological years 1986 to 2000 are used for the validation study because the validation is carried out against an historical data set. Because the hydrological data are available on a daily basis, the effects of weather variability can be taken into account. The GONAT/ANIMO model calculates on a 10-day basis.

### Validation Data Set

Data obtained in three monitoring networks in the Netherlands have been used to validate the STONE model. Two data sets contain data on nitrate concentrations in the upper groundwater for both agricultural and nonagricultural soils, and one contains data for nutrient emissions to surface water.

The first data set contains field observations of nitrate concentrations in the upper groundwater under agricultural soils. These data are taken from 140 different farms throughout the Netherlands during the period 1992 to 1995[8]. In this study,

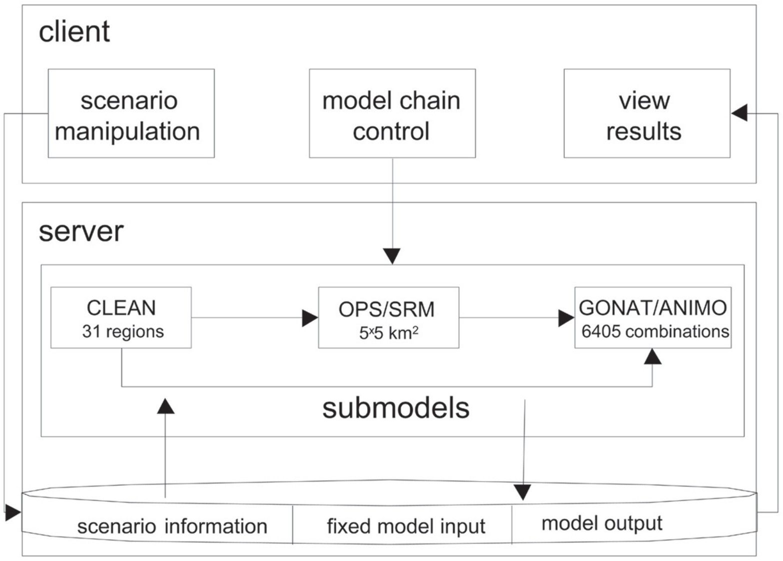

**FIGURE 1.** STONE model layout.

only farms on sandy soils have been considered (100 farms). These regions are especially sensitive to nutrient losses. The available data contain both point observations (about 13,000 nitrate checks) and farm average nitrate concentrations, which are derived from bulk samples. In the farm average values, extreme concentrations are smoothed. The sampling period of the monitoring network runs from April to September. Observed concentrations were generally lower in spring than in autumn. The data set contains two dry (1992 and 1993) and two wet years (1994 and 1995). The groundwater measurements were performed in a depth range of 1 m. The depth of the top of the measurement was always just above the average lowest groundwater level. The majority of the sampling locations have an average highest groundwater level, which is lower than 40 cm under surface level.

The second data set, consisting of field observations of nitrate concentrations in the upper groundwater of sandy soils, contains information about nonagricultural soils, mainly under forest[9]. The data set is made up of 155 point samples taken from October 1989 to April 1990. The groundwater measurements were performed in a depth range of 1 m into the upper groundwater. The depth of the top of the measurement was always below the average lowest groundwater level.

Measurements from the Commission for Integral Water Management (CIW) data set were used for the validation of emission of nutrients toward surface waters[10]. A subset with 141 point observations for total P and 121 point observations for total N was used only with those measurements influenced by agriculture. Measurements were taken from 1986 to 1999, but only during the winter period (October through March). Only sampling locations from the validation data set containing more than three measurements during one winter period were used. Average concentrations were taken for these observations.

## RESULTS AND DISCUSSION

### Validation of Nitrate Concentrations in Upper Groundwater

The STONE results for agriculture were compared to the observed nitrate concentrations in shallow groundwater. Only the agricultural plots on sandy soils with an average highest groundwater level deeper than 40 cm below surface level have been taken into account. The resulting data set covers about 27% of the agricultural land in the Netherlands. Because observed data represent concentrations for an entire year, the annual average of simulated data was taken for comparison. Only the simulated data from 1992 to 1995 were considered for the comparison. An average concentration was calculated for a 1-m column extending from 50 cm above the average lowest groundwater level to 50 cm below the average lowest groundwater level. This depth range served as the depth range for the observations.
Cumulative frequency distributions were plotted to compare observed data with the simulated data from the STONE model (Fig. 2). About 20% (2$^{nd}$ percentile) of the concentrations in the simulated data set have values lower than 50 mg/l (the current groundwater standard). The frequency distribution did not change when concentrations were weighted proportionally to the STONE plot area.

The frequency distributions of the simulated data compare fairly well with the observed data, but the greatest discrepancy between observed and simulated data is found in the lower tail of the cumulative frequency distribution.

Both the farm average values and the point observations are plotted for the observations. As expected, the farm average observations show a steeper distribution. In farm averages, differ-

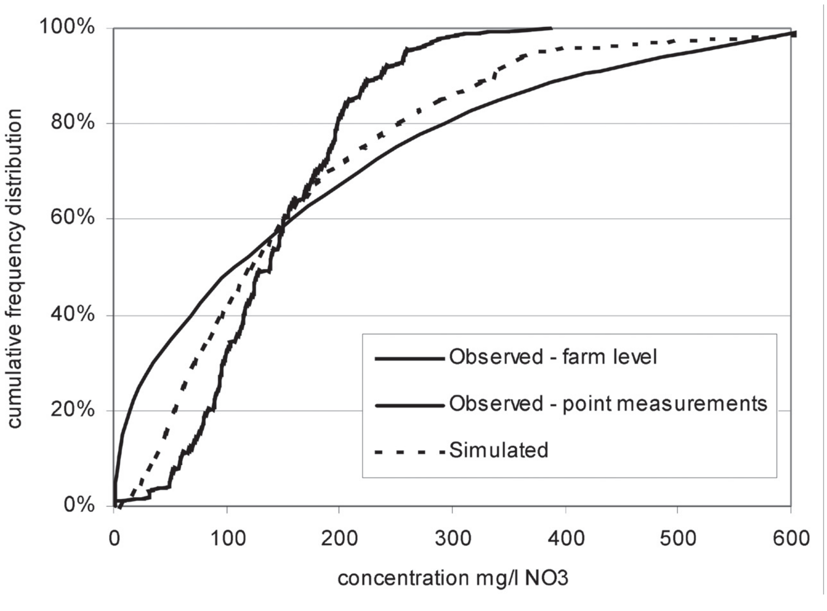

**FIGURE 2.** Frequency distribution of nitrate concentrations in the upper groundwater (1992 to 1995) below agricultural land covering sand, field data (n = 100, n ≅ 13,000) vs. model results (n =1743).

ences between crops, and high and low observations, are smoothed. The frequency distribution of the STONE predictions is found between the frequency distribution of the point scale observations and the farm-level averages. This is also true for the average and median concentrations (Table 1). This is not entirely in line with the above observation that frequency distributions should rise with larger scales, such as that of a STONE plot. A STONE model plot may be larger in size than a farm, but is by definition more homogeneous with respect to its biophysical characteristics. Such homogeneity is never found under real world conditions, i.e., on a farm. The heterogeneous conditions there are leveled off by averaging composite samples. Thus the frequency distributions of the STONE model results may not be as high as the average values found for farms.

It is clear here that the comparison of STONE results with observed data is a complex one and the issue of scale in model validation studies is important[11,12]. Scale differences form the subject of the following stages of the model validation study.

A similar approach was followed for nonagricultural land. For the validation of STONE model results on nonagricultural lands, only the plots that are characterized by nature on sandy soils have been taken into account. The resulting data set covers about 13% of the Netherlands and about 78% of all nature soils. Only data from October 1989 to April 1990 have been taken from the STONE data set. An average concentration was calculated for a 1-m column extending from the average lowest groundwater level to 1 m below this level. Again, this is the depth range serving as the depth range of the observations. Simulated and observed data compare well on cumulative frequency level (Fig. 3).

## Validation of Nutrient Emission to Surface Water

For the validation of nutrient emission to surface water, only the results from the agricultural STONE plots in the direct surroundings of the measurements were used. It has been assumed that only these plots will influence the surface water in which the measurements were made. Other point sources of N and P and

**TABLE 1**
**Average and Median Values of Nitrate Concentrations in the Upper Groundwater Below Agricultural Lands on Sand, Observed vs. Simulated (in mg/l $NO_3$)**

| | Average | Median |
|---|---|---|
| Observed 1992 to 1995 farm level, n = 100 | 145 | 139 |
| Observed 1992 to 1995 point measurements, n ≅ 13,000 | 166 | 107 |
| Simulated by STONE 1992-1995, n = 1743 | 156 | 122 |

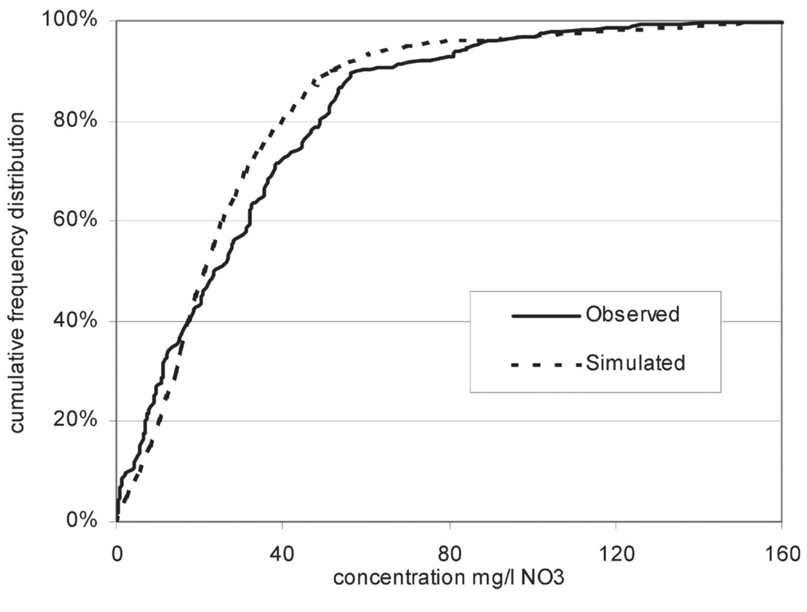

**FIGURE 3.** Frequency distribution of nitrate concentrations in the upper groundwater below nature on sand (1989 to 1990), field data (n = 155) vs. model results (n = 890).

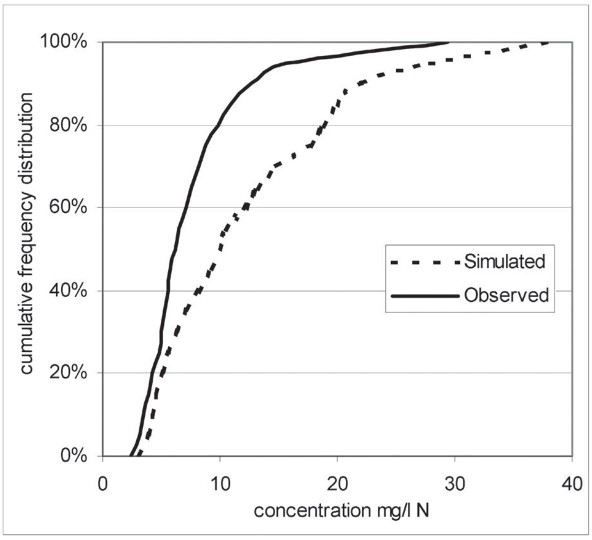

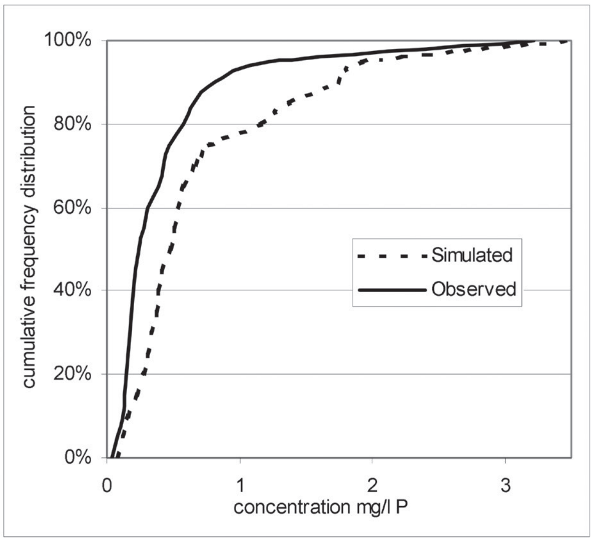

**FIGURE 4.** Frequency distribution of N concentrations in the emission towards surface water from agricultural lands (1986 to 1999), field data (N: n = 121, P: n =141) vs. model results (N: n = 119, P: n = 138).

upstream effects have not been taken into account. Only results from the winter periods have been considered. A concentration of total N was obtained by dividing the sum of the $NO_3$, $NH_4$, and organic N flux by the total water flux toward the surface water. Inorganic and organic P has been added up to obtain the concentration of total P. Because only data from the winter months were used, several temperature-sensitive processes, like uptake by water plants, did not have to be taken into account. Differences between years, and land-use or soil-type differences, have not been considered.

Cumulative frequency distributions were plotted for a comparison of the observed data with the simulated data from the STONE model (Fig. 4). Simulated average concentrations occur about 50% higher than observed values for both P (0.7 vs. 0.4 mg/l) and N (12.0 vs. 7.6 mg/l). For N, the difference is larger for higher percentiles (the $9^{th}$ percentile of the concentration is 12.8 mg $l^{-1}$ for the field data and 22.1 mg $l^{-1}$ for the model results).

The simulated values are expected to be higher than the observed values. This is because the simulated values are concentrations in the flux towards the surface water, whereas observed values are measured in the surface water. Several retention processes can still take place between these two locations. Unfortunately, little is known about the extent of differences that can be expected.

## CONCLUSIONS

The STONE model for the emission of nutrients to groundwater and surface water was developed to evaluate the environmental impact of abatement policies. The first stage of the validation of STONE was carried out in this study, which consisted of validation against lumped observations. Results indicate whether the model can reproduce measured observations, but present only limited information about underlying processes, spatial patterns, or temporal dynamics.

The time and spatial aspects of the observations were considered when extracting data from the model results. Cumulative frequency distributions were plotted for a comparison of the observed data with the simulated data from the STONE model. STONE model results of nitrate concentrations in the upper groundwater compare fairly well with observations from monitoring networks in sandy regions. This is true for both agricultural and nonagricultural land. Both point-scale observations and farm average concentrations have been compared for agricultural land. The model results in concentrations between point observations and farm average concentrations. The fact that the farm average values showed a steeper frequency distribution than the model shows the issue of scale in model validation studies to be an important one. In future validation studies, the observations should be scaled up to the model scale.

STONE model results of nutrient emissions to the surface water also compare fairly well with observed values. Simulated values are expected to be higher than observed values. Simulated values are about 50% higher than observed values for both P and N.

| | | |
|---|---|---|
| Received: | July | 27, 2001 |
| Revised: | September | 17, 2001 |
| Accepted: | October | 3, 2001 |
| Published: | November | 17, 2001 |
